# Diabetic Macular Edema Pathophysiology: Vasogenic versus Inflammatory

**DOI:** 10.1155/2016/2156273

**Published:** 2016-09-28

**Authors:** Pedro Romero-Aroca, Marc Baget-Bernaldiz, Alicia Pareja-Rios, Maribel Lopez-Galvez, Raul Navarro-Gil, Raquel Verges

**Affiliations:** ^1^Ophthalmology Service, University Hospital Sant Joan, Institut de Investigacio Sanitaria Pere Virgili (IISPV), University of Rovira & Virgili, Reus, Spain; ^2^Department of Ophthalmology, Retina Section, Hospital Universitario de Canarias, Tenerife, Spain; ^3^Department of Ophthalmology, University Hospital Valladolid, Ocular Diabetes Unit of IOBA, Valladolid, Spain

## Abstract

Diabetic macular edema (DME) can cause blindness in diabetic patients suffering from diabetic retinopathy (DR). DM parameters controls (glycemia, arterial tension, and lipids) are the gold standard for preventing DR and DME. Although the vascular endothelial growth factor (VEGF) is known to play a role in the development of DME, the pathological processes leading to the onset of this disease are highly complex and the exact sequence in which they occur is still not completely understood. Angiogenesis and inflammation have been shown to be involved in the pathogenesis of this disease. However, it still remains to be clarified whether angiogenesis following VEGF overexpression is a cause or a consequence of inflammation. This paper provides a review of the data currently available, focusing on VEGF, angiogenesis, and inflammation. Our analysis suggests that angiogenesis and inflammation act interdependently during the development of DME. Knowledge of DME etiology seems to be important in treatments with anti-VEGF or anti-inflammatory drugs. Current diagnostic techniques do not permit us to differentiate between both etiologies. In the future, diagnosing the physiopathology of each patient with DME will help us to select the most effective drug.

## 1. Introduction

Diabetes mellitus (DM^*∗*^ all acronyms are given in a summary list at the end of the text) is a worldwide pandemic disease. As of 2010, more than 200 million people had been diagnosed with diabetes, and this number is predicted to increase by 62% by 2025 [1]. This increase is due to an increase in obesity together with the increased life expectancy of the world population. DM complications include macroangiopathy (myocardial infarction or vasculocerebral stroke) and microangiopathy (diabetic nephropathy, neuropathy, and retinopathy).

Diabetic retinopathy (DR) is the most common cause of blindness in Europe [2], affecting 1.9% of patients with DM [3]. Furthermore, 2.64% of diabetic patients have visual sight-threatening diabetic retinopathy (STDR). The major cause of visual impairment in DM patients is diabetic macular edema (DME), with an annual incidence of 2.19%. DME is a consequence of DR in the macular area and is secondary to retinal barrier rupture, which is in turn secondary to a range of metabolic changes brought about by hyperglycemia [4]. The most important molecule in retinal barrier rupture is the vascular endothelial growth factor (VEGF). The introduction of anti-VEGF and steroid drugs for treating DME has changed our knowledge of pathophysiology [5]. But the uses of anti-VEGF drugs have revealed that around 30% of patients are resistant to intravitreal treatment [6].

The fact that so many patients are proving to be resistant to treatment would suggest that other physiological mechanisms must be involved. A low-grade inflammatory process has been shown to be a possible cause, which would explain the improvement in DME after steroid intravitreal injections have been administered. Angiogenesis and inflammation have been shown to be involved in the pathogenesis of this disease, but it still needs to be clarified whether angiogenesis following an overexpression of VEGF is a cause or a consequence of inflammation. This review aims to update our knowledge on the etiology of DME, which will help us to understand the differences between the vasogenic and inflammatory etiology of DME.

In a series of questions and answers, we describe the current knowledge about DME and its etiology.

## 2. What Is the Tissue Target of Diabetic Macular Edema?

**Figure 1:**
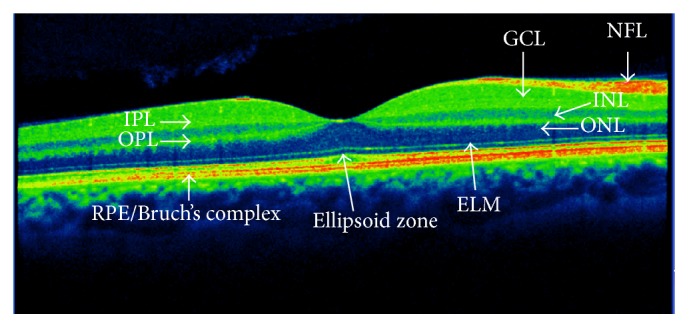
Optical coherence tomography of the macular area, description of different layers: RPE/Bruch's complex: retinal pigment epithelium and Bruch's layer; ELM: external limiting membrane; ellipsoid zone: inner segments/outer segments of photoreceptors (ellipsoid layer); ONL: outer nuclear layer (photoreceptors); OPL: outer plexiform layer; INL: inner nuclear layer (bipolar cell); IPL: inner plexiform layer; GCL: ganglion cell layer; NFL: nerve fiber layer.

The retinal macular zone is the target of diabetic macular edema (DME). In order to understand the anatomic changes in DME. We describe the retinal anatomy with reference to optical coherence tomography (OCT), a technique used currently in the diagnosis and follow-up of DME, which allows us to see the multiple layers of the retina and choroid. We describe these different layers according to the classification laid down by the international panel of experts in vitreoretinal diseases [7] (Figure 1).

### 2.1. Retinal Anatomy

The retina is a complex structure of neural tissue made up of different cell types. The retina is divided into the* neurosensory retina (NR)* and the* retinal pigment epithelium (RPE)*. The NR includes all layers from the photoreceptors to ganglion cells, and the RPE is a monolayer formed by a single cell type located in the outermost part of the retina (Figure 1).

#### 2.1.1. Nonsensory Layer of the Retina

- The* retinal pigment epithelium (RPE)* is a monolayer of cells characterised by a large presence of melanin pigment in the cytoplasm. This allows it to absorb light, which then reaches the retina. RPE is a multifunctional layer. The apical cell of the RPE is closely linked to the photoreceptors, which form a true functional unit. In the other side, the RPE forms a complex with Bruch's membrane seen at optical coherence tomography (OCT) denominated as RPE/Bruch's complex.

#### 2.1.2. Neurosensory Retinal Layers

(*α*) The* photoreceptor layer* is formed by rods and cones in its internal and outer segments (IS/OS) and includes two layers observable with OCT, both of which are important in visual acuity impairment:
  - *Ellipsoid Zone*. This is located just above the RPE and is formed by the union of the IS/OS of the photoreceptors. In the OCT, the sagittal slice of the retina is shown as a hyperreflective line. In several functional studies of the macula, a link has been found between the integrity of this layer and resulting visual acuity.
  - *External Limiting Membrane Layer (ELM)*. This is seen as a discrete line, located just above the ellipsoid zone, which separates the photoreceptor nucleus from its internal segments. It comprises the apical processes of Müller cells and is similar to the ellipsoid zone; there is a link between the integrity of this layer and visual acuity.
(*β*) The* outer nuclear layer (ONL)* is formed by the cell nuclei of rods and cones. The cones are responsible for visual acuity and colour perception.
(*χ*) The* outer plexiform layer (OPL)* is established by the synapses of bipolar cells between photoreceptors. In addition, it includes horizontal interneuron cells that adjust vision in extreme environmental light conditions.
(*δ*) The* inner nuclear layer (INL)* contains the nuclei of bipolar cells. Bipolar cells are the first-neuron cell to process the electrical stimulus coming from the photoreceptors before transmitting it to the ganglion cells. These cells are responsible for the electrical response of the retina as objectified in multifocal electroretinography.
(*ε*) The* inner plexiform layer (IPL)* is composed of synapses between the bipolar, ganglion, and amacrine cells (responsible for adjusting the retinal image).
(*ϕ*) The* ganglion cell layer (GCL)* contains ganglion cells which are the second-neuron cell in visual via and comprises cells that transmit impulses from the photoreceptors through their long axons to the thalamus.
(*γ*) The* nerve fiber layer (NFL)* is formed by the ganglion cell axons.

The edema is intracellular at the beginning, with Müller cells swelling as the first affected cells. Its progression induces its apoptosis. Other cells such as bipolar cells, ganglion cells, and photoreceptors undergo presynaptic elongation and a reduction of its prolongation until the edema is reversible if metabolic status ameliorates. After this phase, the liquid passes through the cell membrane and accumulates in the interstitial space, forming cysts. In DME, cyst formation appears in the inner layers with little cysts that progress to the external layers forming much larger cysts, which will become visible under retinal biomicroscopy and fluorescein angiography. At the external plexiform layer, the edema allows lipid deposition as hard exudate. The rupture of retinal pigment epithelium allows liquid accumulation under the neurosensory retina and its detachment, which is a form of edema described in the classification of optical coherence tomography.

## 3. What Is the Role of Retinal Barriers in Diabetic Macular Edema?

The eye, like the brain, has mechanisms that hinder the passage of certain substances or microorganisms, thereby reducing the risk of inflammation. This is achieved by different barriers, which include

- the retinal pigment epithelium (RPE), which acts as an external-blood retinal barrier (e-BRB);
- the retina vascular plexuses, which acts as an inner-blood retinal barrier (i-BRB);
- the endothelial cells of capillaries and pigmented epithelium of the iris, forming the anterior hematoaqueous barrier;
- the nonpigmented epithelium of ciliary processes, that forms the posterior hematoaqueous barrier.

The last two barriers form anterior segment barrier influencing the composition of the aqueous humour, with special characteristics different from retina interstitial liquid. Since, in this review, we do not analyse the anterior segment barriers, a description of the epithelium of ciliary processes or the endothelial cells of capillaries of the iris and ciliary muscle has been excluded. Rather, our discussion focuses on the posterior segment barriers: the external-blood retinal barrier (e-BRB) and the inner-blood retinal barrier (i-BRB). The blood-retinal barrier keeps the retina isolated from intravascular molecules, and the RPE acts as an external barrier via its tight cell junctions.

### 3.1. External-Blood Retinal Barrier (e-BRB) and Retinal Pigment Epithelium (RPE)

The RPE is the external retinal layer whose functions include the following:

- Regeneration of all-trans-retinol to 11-cis-retinal is a process essential for vision, which supplies vitamin A and glucose to the photoreceptors.
- Phagocytosis of the external disc and of the old photoreceptors is degraded during the visual cycle.
- Nutrition functions allow oxygen and nutrients to pass from the choriocapillaris to external segments of the retina.
- The RPE is in contact with the choriocapillaris layer of the choroid, which allows the diffusion of molecules into the RPE. The cells of the RPE have strong intercellular junctions between them that act as an external-blood retinal barrier (e-BRB), determining the exchange of controlled substances between the neurosensory retina and choriocapillaris. The RPE is important for pumping fluid into the choriocapillaris, preventing the formation of a macular edema [8].

Despite RPE appearing to act as an independent external barrier, such a separation from i-BRB is not clear. In fact, most of the molecules secreted by the two barrier cell components act simultaneously. The RPE synthesizes various molecules, such as the vascular endothelial growth factor (VEGF) and pigmented epithelium-derived factor (PEDF). The VEGF causes increased vascular permeability alongside its known angiogenic effect, while the PEDF has an antagonistic effect.

### 3.2. Inner-Blood Retinal Barrier (i-BRB)

The capillaries of the central retinal artery provide a blood barrier that protects the retina from potentially harmful molecules. The retinal capillaries have two different plexuses: the superficial capillary plexus located within the ganglion cell layer (GCL) and the capillary plexus located within the inner nuclear layer (INL), adjacent to inner plexiform layer (IPL) and synaptic portion of the outer plexiform layer (OPL).

The two capillary plexus endothelial cells of the retina are strongly joined by tight and adherents junctions [9], each of which is composed of different molecules (Table 1).

The transport of molecules requires two routes:

1. *Paracellular route* includes passage through the endothelial unions, which vary during paracellular transport, opening and closing according to the demands of the tissues of small molecules and solutes.
2. *Transcellular route* includes a vesicular transporter which exists across the endothelial cells and is selective and regulated by membrane cell transporters.

i-BRB also includes different structural cells around the endothelial cells, which aids barriers, such as pericytes, macroglial cells like astrocytes, and Müller and microglial cells. In addition, the pericytes and endothelial cells are surrounded by a basal cell membrane that contributes to i-BRB.

In diabetes, the two barriers suffer a metabolic disruption due to different molecules being formed in a hyperglycemic environment [10].

## 4. What Are the Metabolic Clues in Diabetic Retinopathy and Macular Edema?

The most important finding in diabetic retinopathy (DR) is the presence of hyperglycemia, which acts on different molecular pathways and damages the blood-retinal barrier, overpowering the cells that make up these structures, like endothelial cells and pericytes.

### 4.1. Glutamate

Is being an important pathway in the development of DR and DME the function of glutamate, a molecule that is present in high levels in diabetes? Glutamate (Glu) is the most important excitatory neurotransmitter in the brain and retina. Various studies have found high levels of extracellular retinal glutamate linked to DR. This excess causes an activation of the sodium-calcium intraneuronal receptor, initiating the mechanisms of apoptosis [11]. Under certain physiological conditions, Müller cells are responsible for regulating the level of glutamate in the extracellular and intracellular compartments. Thus, when producing excess level of extracellular glutamate, the Müller cells are internalised, facilitating its homeostasis. In diabetes, this mechanism is weaker due to its excess, producing cell toxicity.

### 4.2. Hyperglycemia

 Hyperglycemia has been considered in recent decades as the main cause of the onset and progression of DME and DR. In this regard, two epidemiological studies, the Diabetes Control and Complications Trial (DCCT) and the United Kingdom Prospective Diabetic Study (UKPDS), have reported that intensive control of glycemia is linked to a lower risk of the onset and progression of DR in both type 1 diabetes and type 2 diabetes patients [12, 13]. However, hyperglycemia does not fully explain the wide range of functional and cellular changes that appear over the course of DR [13]. Clinical experience has shown that there is a group of diabetes patients who are unable to prevent the onset or progression of DR and DME despite achieving good metabolic control of the disease. Hyperglycemia maintained over time produces an enzymatic glycation of proteins or advanced glycation end-products (AGEs). All AGEs are highly prevalent in the retinal vasculature of diabetes patients and are involved in microvascular and macrovascular complications [14, 15]. AGEs provoke changes in the extracellular matrix (ECM) and an increase in vascular stiffness. Moreover, and more importantly, they stimulate the endothelial membrane receptors of advanced glycation end-products (RAGE), setting into motion various metabolic pathways (signalling pathways) that eventually increase the expression of molecules such as inflammatory intercellular adhesion molecule-1 (ICAM-1) and VEGF, as well as a synergistic decrease of nitric oxide (NO), causing oxidative stress in pericytes and leading to their apoptosis. The result of this process is capillary vasoconstriction, increased leukocyte adhesion resulting in hypoxia, and retinal capillary hyperpermeability with DME [16].

### 4.3. Results of Hyperglycemia in Diabetic Patients

**Figure 2:**
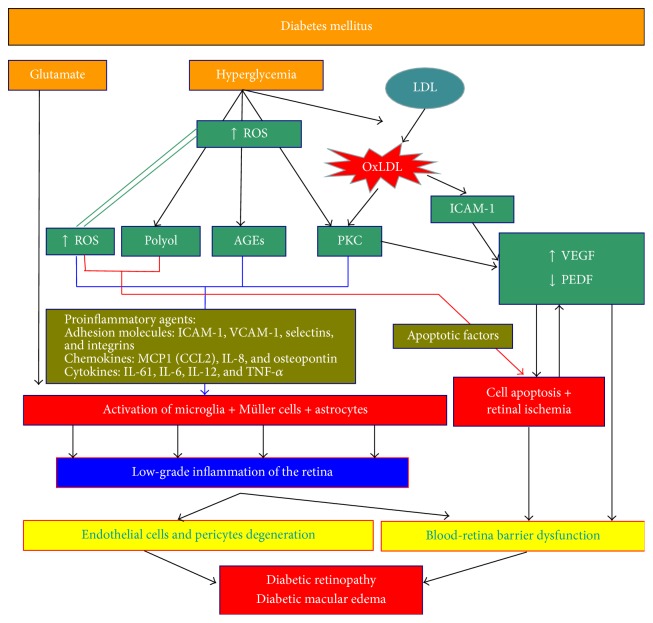
The different pathways in the development of diabetic macular edema.

Hyperglycemia induces different, imbricated metabolic pathways, initiating the development of a cascade that culminates in the development and progression of diabetic retinopathy (Figure 2).

- Increase in polyol production is an implicated pathway in retinal neurodegeneration. In the first step, glucose is converted into sorbitol by the action of aldose reductase; in the second step, sorbitol is converted into fructose. The sorbitol remains in the intracellular space, inducing cellular damage by an unknown pathway. In parallel, the activation of the aldose-reductase enzyme via excess of glucose induces the downregulation of glutathione, which is an antioxidant, subsequently increasing oxidative stress. The two mechanisms (sorbitol and aldose-reductase enzyme pathways) are produced into the mitochondria and they increase the oxidative stress induced by the polyol pathway, which in turn damages retinal cells and induces DR.
- The formation of AGEs alters the transmembrane proteins of i-BRB and initiates the inflammatory cascade. AGEs are the result of glucose-membrane cell protein unions, especially the union of fructose and membrane cell protein, and are named as glycation process. Glycation with fructose occurs at a higher rate than with glucose alone; in diabetic patients, excess of fructose permits the formation of a great number of AGE molecules. AGEs produce cell damage through their union with RAGE, and oxidative stress increases its expression, increasing the effect of AGEs. The AGE-RAGE complex induces various diabetic vascular complications, including proinflammatory responses.
- The activation of protein kinase C (PKC) is an important pathway in the disruption of i-BRB. PKC is one of the members of the kinase family, which is implicated in phosphorylation reactions. Overaction of PKC occurs in oxidative stress via the formation of excessive diacylglycerol (DAG), which upregulates PKC activation. PKC is important in different intracellular functions, including immunoresponses, cellular growth and development, and guiding transcription at the membrane cell level. PKC overaction causes an upregulation of these activities and initiates cell growth inducing angiogenesis. Other events induced directly by PKC or by increased expression of different factors such as VEGF or transforming growth factor-beta 1 (TGFB1) include the accumulation of extracellular matrix, fibrinolysis, and inflammatory responses.
- Oxidative stress, secondary to the accumulation of free radicals in the form of reactive oxidative species (ROS), is linked to histopathological changes such as the thickening of the basement membrane and the loss of endothelial cells and pericytes. The accumulation of any advanced glycation end-products (AGEs) increases the production of ROS. The polyol pathway decreases the production of the antioxidant glutathione, which inhibits ROS. Finally, ROS increases the activity of protein kinase C (PKC). The interaction between ROS and the three previously described pathways under the double route of ROS pathway activation defines ROS as playing a key role in DR development, which is difficult to control. Mitochondrial dysfunction, the source of oxidative stress, can be a potential target for DR treatment.

### 4.4. Protective Retinal Metabolites

The retina is a neural tissue which, like the brain, synthesizes a series of neurotrophic factors necessary for homeostasis. Its role is largely to neutralise the increased oxidative stress that occurs in certain circumstances; for example, the brain-derived neurotrophic factor (BDNF) is synthesized in both neurons and glial cells of the retina. Some studies have shown that it protects the retina and optic nerve from ischemia. BDNF has been shown to stimulate glutamate uptake by Müller cells [17] and the factor derived from the pigment epithelium-derived factor (PEDF) to protect cells from excess of glutamate [18]. DM disturbs the relationship between the neuroprotective, antiangiogenic, and proangiogenic factors. Among neuroprotective factors, a decrease in the BDNF and ciliary neurotrophic factor (CNTF) has been reported, as well as a decrease in the factor derived from the PEDF. At the same time, there is an observed increase in VEGF, which plays an important role in the onset and progression of DR and DME and protects retinal ganglion cells. This shows that there is a dual effect where retinal ganglion cells are protected and i-BRB is disrupted.

## 5. What Are the Clues to the Rupture of the Inner-Blood Retinal Barrier?

In DR and DME, there is an imbalance between proangiogenic and antiangiogenic factors in favour of the former, with higher levels of VEGF, platelet-derived growth factor (PDGF), angiopoietin-2 (Ang-2), osteopontin (OPN), and erythropoietin (EPO). At the same time, a decrease in PEDF, endostatin (ES), and angiostatin (AS) has also been observed.

The most important factor in angiogenesis in retinal tissue is VEGF, which is involved in the genesis of proliferative DR and DME. VEGF is a molecule present in the vitreous of patients with RD and DME, with its levels being proportional to the severity of the diseases. On average, the level of VEGF in patients with DME or DR is 10 times higher than in diabetes patients, without affecting the retina. Various cells produce and synthesize VEGF:

- Müller cells
- Lymph nodes
- Glial cells
- Retinal pigment epithelium
- Endothelial cells
- Pericytes

High levels of VEGF increase the expression of the inflammatory intercellular adhesion molecule-1 (ICAM-1), leading to retinal capillary leukostasis and important pathogenic factors of diabetic microangiopathy [19–22].

PEDF is a glycoprotein produced by the RPE that inhibits angiogenesis. Funatsu et al. [23] found lower levels of this factor in the vitreous of patients with DME. It seems that the balance between VEGF and PEDF is essential for the maintenance and integrity of i-BRB, thus regulating its permeability [24]. In addition, the PEDF blocks the binding of VEGF to its receptor, reducing its action.

Another factor implicated in DR is the platelet-derived growth factor (PDGF), which is secreted by endothelial cells and is responsible for the function of pericytes [25]; a reduction in its function produces and increases the secretion of *α*-tumour necrosis factor (*α*-TNF), which in turn increases ICAM-1 expression.

The most accepted hypothesis to explain the increased expression of VEGF in the vitreous of patients with DR and DME is that retinal hypoxia is caused by an obstruction and loss of retinal capillaries. The capillary blockage is due to the adhesion of leukocytes and endothelial cells, which is facilitated by an increased presence of the ICAM-1 on the endothelial surface [21]. It is believed that the increased expression of the ICAM-1 protein is produced in response to the increased exposure to VEGF receptor activation via AGE protein induction.

The hypothesis that retinal ischemia leads to increased VEGF is congruent with the argument that increased expression of VEGF is regulated by hypoxia-induced factor 1*α* (HIF-1*α*). In hypoxic conditions, there is a reduction in HIF-1*α*, which results in the activation of genes that in turn produce proangiogenic factors (VEGF, Ang-2, etc.), [26, 27]. Studies have found high levels of HIF-1*α* and VEGF in the vitreous of patients with proliferative DR and DME [28].

In summary, VEGF, which is higher in the vitreous of DR patients, is the most important molecule in the development of changes in the vascular bed, the rupturing of the blood-retinal barrier, and the induction of angiogenesis [29].

## 6. What is the Pathogenesis of Diabetic Macular Edema?

### 6.1. Cytotoxic and Vasogenic Edema

In macular edema, liquid accumulation can occur in intracellular or extracellular spaces. Edema-induced accumulation of liquid within the intracellular space is defined as cytotoxic, while accumulation of liquid in the extracellular space is defined as vasogenic edema. These two different edemas can appear in different pathologies: in arterial occlusion by emboli, the edema is located in the intracellular space and is visible in OCT as hyperreflectivity in retinal nuclear layers; in venous occlusions, the vasogenic edema is the most important form.

In patients with DME, the two forms of edema appear: cytotoxic form at the beginning and a vasogenic form later on.

Cytotoxic form can result from increased sorbitol, lactate, and phosphates in the intracellular space, secondary to hyperglycemia.

Vasogenic form can be produced by many of the molecules described previously, including VEGF, nitrous oxide, and free radicals, which produce a rupture of i-BRB. The amount of accumulated extracellular fluid is determined by the difference between the osmotic and hydrostatic pressure in the retinal veins and arterioles against the extracellular environment [7, 30]. The result of vasogenic edema is an accumulation of fluid, mainly in the extracellular layer of the external plexiform and the inner and outer nuclear retinal layers. In some patients, a detachment of the neurosensory retina occurs.

The presence of cytotoxic and vasogenic lesions in DM patients induces the reduction of pericytes, Müller cells, and astrocytes, with an increase of basal membrane capillaries and a decrease in the number of endothelial cells, which in turn induces the hyperpermeability of retinal vessels due to the rupture of i-BRB. This rupture must occur before we can observe clinical signs of DR.

### 6.2. The Importance of Müller Cells

Müller cells are the most important glial cells in the retina. Located alongside the layers of the neurosensory retina, Müller cells' nuclei are in the internal nuclear layer, and their extensions (axons and dendrites) contact all nuclei of retinal cells. Müller cells permit contact between retinal cells and different compartments: vitreous, retinal vessels, and the subretinal space. They also have ionic channels, transmembrane proteins, and different enzymes. An important characteristic of Müller cells is their great conductance for potassium (K^+^).

The functions of Müller cells are

- elimination of extracellular liquid into the retinal vessels;
- regulation of blood flux;
- relationship with glucose metabolism of retinal neurons;
- maintenance of retina pH by aqueous homeostasis;
- production of glutamate, which is implicated in neuronal transmission.

Macular edema appears as a consequence of an imbalance between the liquid passing from retinal vessels to the extracellular space and the reabsorption of the liquid from the extracellular space into the vessels. Müller cells are responsible for the active transport of liquid into retinal vessels; in DM, Müller cell metabolism is disturbed, and a dysfunction of interstitial liquid homeostasis and potassium exists, which permits the intracellular accumulation of liquid with increased difficulty discharging the liquid into the vessels; in the first step, cellular swelling occurs, leading to cell rupture and an increase of liquid in the extracellular space, which developed cysts spaces. This finding is in accordance with the steroid effect over these channels, which restores its metabolic functions, helps restore potassium, and promotes aqueous drainage from the retina into the vessels.

### 6.3. Diabetic Retinopathy Is Secondary to Microangiopathy: Is There a Concomitant Neuropathy?

DR and DME are currently considered manifestations of parallel vascular and neuronal degenerative processes. Several studies support this view, suggesting that the existence of subclinical neuronal impairment prior to the appearance of vascular lesions is typical of DR.

Tests that evaluate the function of the retina, such as multifocal electroretinograms [31], contrast sensitivity tests, or automated perimetries, have highlighted the vulnerability of retinal cells. The result is an increase in retinal neuronal apoptosis [32] together with changes in retinal vasculature that culminate in the compromising of vision in diabetes patients.

In physiological conditions, a close relationship exists between the cellular and vascular component of the retina in order to maintain the homeostasis necessary for normal operation. This interaction causes neurovascular damage in the course of retinal degeneration via changes that induce early microvascular changes, events that involve the breaking of the blood-retinal barrier, which is the stage prior to the onset of DME [33].

It is not known what causes neuroinflammation in the retina. However, various nociceptive stimuli are involved, such as hyperglycemia, glutamate, deregulation of neurotrophic retinal factors, oxidative stress, AGEs, and stress-level endothelial reticulum [34]. There are those who consider DR to be a manifestation of a systemic inflammatory condition of DM.

Regardless of which factors cause inflammation of the retina, it seems that it is caused by the activation of the immune system of the retina itself, especially the microglial cells.

Microglial cells, derived from monocytes as perivascular macrophages, modulate the immune response, but, in situations of chronic stress, their inflammatory capacity increases. Under normal conditions, microglial cells are located at the two plexiform layers and are responsible for the control of the extracellular compartment of the retina, maintaining tissue homeostasis and playing a role more similar to neurons, judging by the highly branched projections they present [35, 36]. Thus, microglial activation is necessary to eliminate pathogens and apoptotic neurons. However, in DR, its activation generates anatomical and functional changes in the retinal cells.

Under conditions of high activation, microglial cells acquire a more amoeboid phenotype with increased motility, and their location is displaced from plexiform layers to the subretinal space. In addition, microglial cells synthesize and remove cytokines, proteases, nitrous oxide, and ROS in the extracellular medium, which causes neuronal death [37]. The subretinal space is an immunoprivileged space that can be altered by the presence of microglial cells and the production of cytokines. In a recent study, porous holes were observed between the retinal pigment layer and the choroid, which facilitate the passage of cellules between both layers. In the same study, Graeber et al. [38] observed, in a murine diabetic model, that, in the case of diabetes, the number of holes in epithelium pigment retina layer (EPR) increased in order to permit the transport of more inflammatory cellules to the choroid space; they also observed an increase of ICAM-1 and caviling-1 (CAV-1), both which are implicated in leukostasis, with the number of holes diminishing after one year. The microglia participate in DR inflammatory response by increasing their number around the vessels and expressing the chemokine C-C motif ligand 2 (CCL2), which induces the recruitment of macrophages to the retina. In the first step, the microglial cells pass into the subretinal space and then migrate to the choroid; but when the EPR layer becomes damaged and cannot permit the passage of more cells, the microglia accumulate at the subretinal space, increasing the inflammatory response.

In sum, it seems that the microglial cells, along with Müller cells and astrocytes, initiate inflammatory processes in the retina, and reactive glial cells amplify this response [39]; and a low-grade inflammation is maintained by the production of cytokines such as interleukin 6 (IL-6) and interleukin 8 (IL-8) or C-C motif ligand 2 [40]. IL-6 alters the function of astrocytes, which give structural support to the capillaries in the retina, thus breaking i-BRB. At the same time, the retina's ability to internalise glutamate from Müller cells is reduced. IL-8 and CCL2 act on neutrophils and monocytes, respectively. Little is known about the migration of white blood cells into the retina, but the role of IL-8 and CCL2 is analogous to the brain in that they attract leukocytes.

These cytokines have been consistently shown to be elevated in patients with DR and DME, correlating positively with severity. Differences in expression profile of cytokines between diabetics, without and with mild DR, and controls have been documented [41]. Also the histology of patients with DR provides evidence of the perivascular infiltration of monocytes into the most affected retinal areas.

## 7. What Is the Current Treatment of Diabetic Macular Edema?

As we described previously, the rupture of i-BRB involves many molecules, the most important of which, VEGF, is found in the vitreous of DM patients and acts on endothelial cells through various mechanisms:

- By increasing vascular permeability due to the formation of fenestration in retinal vessels
- By increasing angiogenesis directly as a potent mitogen of endothelial cells and indirectly by stimulating metalloprotease production and inhibiting metalloprotease inhibitors
- By acting as a proinflammatory agent

**Figure 3:**
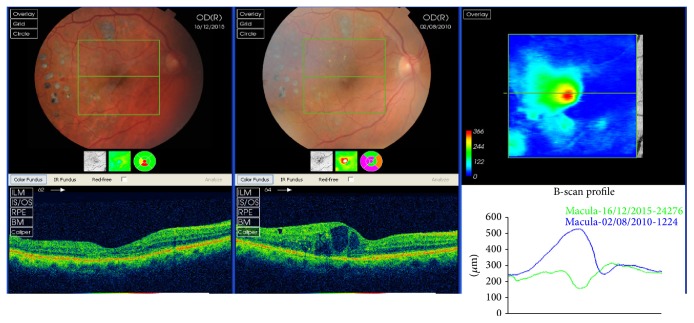
Evolution of a patient with diabetic macular edema, from 2012 to 2015. The patient did not respond to anti-VEGF but to steroids. Perhaps, the hyperreflective retinal spots, seen in the first tomography in the outer plexiform layer temporal to the fovea, can be a predictive sign of a positive response to steroids.

Pathologically, VEGF production is altered during DR and DME, secondary to hyperglycemia, PKC activation, and AGE protein production. Currently, we use therapies based on VEGF inhibitors (blocking or inhibiting VEGF) in the management of DME (Figure 3), which helps prevent i-BRB disruption [42–44].

Since 2010, anti-VEGF treatments have been highly effective against DME, reducing central macular thickness (CMT) in patients with edema. Despite no direct relationship having been observed between a decrease in CMT and the recovery of visual acuity, all ophthalmology guidelines recommend a decrease in CMT as a target for the resolution of DME.

The first two multicentre studies, RESOLVE [45] and RESTORE [46, 47], have reported that anti-VEGF treatments are superior to laser treatments. In the RESOLVE study, the safety and efficacy of ranibizumab in DME were investigated in a 12-month, multicentre, controlled, double-masked study that randomized patients into three groups: two groups with different concentrations of intravitreal ranibizumab and a group with a placebo injection at twelve months. Mean visual acuity increased in the two ranibizumab groups, with no significant differences between them, and decreased in the control group, concluding that ranibizumab is effective and safe in DME treatment. For DME in the RESTORE study, ranibizumab as a monotherapy or combined with laser versus monotherapy with laser was administered during also a 12-month, randomized, double-masked, multicentre phase III study, but the study included photocoagulation laser as an alternative treatment. Patients were classified into three groups: ranibizumab and laser, or placebo injections and laser. The authors conclude that ranibizumab, both as a monotherapy or combined with laser, improves visual acuity more than standard laser does in patients with visual impairment due to DME. In the RESTORE study at three years, 47 patients were eligible to receive individualized ranibizumab treatment as of month 12, guided by visual acuity and disease progression criteria at the investigators discretion. The study concludes that ranibizumab is effective in improving and maintaining visual acuity and central retinal thickness with a progressively declining number of injections over 3 years of individualized dosing.

Results showed that ranibizumab was effective in improving and maintaining visual acuity (VA) and CMT outcomes, with a progressively declining number of injections needed over a 3-year treatment regimen. Intravitreal injections were generally well tolerated, with no safety concerns being reported over the three-year study period apart from frequently reported cataracts in 16.3% of patients [47].

Since the Diabetic Retinopathy Clinical Research (DRCR) network study [48], we have the possibility of using steroids. The study compared 4 mg and 8 mg intravitreal doses of triamcinolone acetonide (IVTA) to focal/grid laser photocoagulation. The 4 mg IVTA group had better visual acuity after three years than the 8 mg IVTA group, but the laser group had better visual acuity than either IVTA group, probably due to the fewer number of complications, cataracts, and glaucoma, which decreased visual acuity in the groups treated by IVTA.

In a second randomized controlled trial by the DRCR network, the focal/grid laser method alone was compared to a 4 mg dose of IVTA and laser. Similar to the previous study, the IVTA and laser performed better than the laser alone in terms of visual acuity but also had increased rates of cataracts and higher intraocular pressure (IOP). In the subgroup analysis of patients who were pseudophakic at baseline, the IVTA and laser group performed better than the laser treatment group, equivalent to the ranibizumab group [49].

Other steroids have also been studied in the treatment of DME, with dexamethasone implants into the vitreous demonstrating effectiveness in resolving DME in refractory cases [50, 51]. Also, fluocinolone acetonide, has also been effective [52] but with a large number of patients still developing cataracts (91% underwent a cataract extraction by the fourth year). In addition, IOP increased by up to 30 mmHg in 61.4% of implanted eyes and 33.8% required surgery for ocular hypertension [53].

### 7.1. When Treating DME, Should We Use Anti-VEGF Drugs or Steroids Drugs?

Despite the effectiveness of anti-VEGF treatment (Figure 3), if the published data is observed carefully, and different conclusions can be reached. In the most recent studies, the RIDE-RISE trials using ranibizumab [54, 55] and the VISTA-VIVD trials using aflibercept [56], visual acuity increased following intravitreal ranibizumab and aflibercept. RIDE-RISE and VIVID-VISTA are two studies that used ranibizumab and aflibercept, respectively. In the first study with ranibizumab, patients were randomized to monthly intravitreal injections of 0.3 or 0.5 mg or placebo. At 24 months, visual acuity had increased and central retinal thickness had decreased in two ranibizumab doses compared to the control group, concluding that ranibizumab is effective. In the second study with aflibercept, patients received 2 mg every 4 weeks, 2 mg every 8 weeks, or a laser control. Results showed that the 52-week visual and anatomic superiority of aflibercept over laser continued through to week 100, with similar efficacy in both aflibercept groups.

Despite the good results in both studies, we observe that only about 38% of patients achieved the targeted increase of 15 or more letters (equivalent to three lines in the optotypes) after treatment; and, in all studies, about 30% of patients were nonresponsive. The DRCR network is funded by the National Eye Institute, and, with the collaboration of 177 clinical sites in the United States and Canada, it has conducted multiple studies about DME treatments. Properly defined, the DRCR network is a collaborative effort dedicated to facilitating multicentre clinical research on DR, DME, and associated conditions.

The DRCR network compared multiple different treatments for DME, including anti-VEGF drugs alone or in combination with lasers or steroids; as we have said, patients with DME were classified as either chronic or nonchronic DME, attending the response to anti-VEGF treatment. Nonresponders or chronic DME is diagnosed if there is a failure in the treatment, which is defined as having no increase in visual acuity over five letters or no 10% reduction in central retinal thickness. These changes must take place after four anti-VEGF intravitreal injections or after six months of treatment with the same responses.

It is interesting to note the latest results published in Protocol T [57, 58]; these studies, published after one- and two-year follow-ups of a clinical series of DME patients, demonstrated that if the initial response to anti-VEGF drugs at three months is good, final visual acuity will be good. These studies divided patients into three groups:

1. Patients with an initial response of no fewer than five letters in the optotypes at three months
2. Patients with an initial response of 5–9 letters
3. Patients with an initial response of more than 10 letters

The first group included about 39.7% of patients in the sample, the second group included 23.2%, and the third group included 37.1%. All three groups retained increased visual acuity along the next 12 months.

The COCHRANE study, which reviewed the effectiveness and safety of intraocular steroids in treating DME (triamcinolone acetonide, fluocinolone acetonide implants, and dexamethasone drug delivery systems), concluded that intravitreal steroid injections may improve visual outcomes in eyes with persistent or refractory DME but with a large number of complications. Since the studies in the review focused on chronic or refractory DME, the question arises as to whether intravitreal steroid therapy could be of value at other stages of DME, especially at earlier stages, either as a standalone therapy or in combination with other therapies, such as laser photocoagulation [59].

## 8. What Are the Examination Techniques of Diabetic Macular Edema?

There are many different techniques for examining the macular area, including biomicroscopy, fluorescein angiography, and optical coherence tomography (OCT).

Biomicroscopy using a noncontact fundus lens is routinely used in clinical practice as the first type of examination of patients with DR, after screening by retinography. However, this technique does not allow us to retain the images and cannot determine how large a retinal lesion is.

The two principal techniques currently used in the diagnosis and follow-up of patients with DME are optical coherence tomography (OCT) and fluorescein angiography (FA). The latter is important for determining the presence of ischemia in the retinal tissue, but it is an invasive technique with possible severe adverse effects as serious angioneurotic reaction in 2% of patients; thus, in clinical practice, OCT is the most used technique in DME follow-up and for determining responses to treatment.

In the two next sections, we explain how to apply these techniques in DME and whether it is possible to determine its vasogenic or inflammatory origin.

### 8.1. Optical Coherence Tomography and the Different Types of DME

The challenge was to determine whether there is a marker in OCT that would help us in the diagnosis of a predominance of vasogenic or inflammatory changes in the macular area. What do we know about the signs of DME in OCT?

Over the last 15 years, the introduction of OCT has revolutionised macular examination. The image of the macula and its layers allows us to diagnose much of its pathology (Figure 1). The follow-up for DME treatment currently includes OCT, and, depending on the results, treatment is either continued or discontinued (Figure 3). Therefore, it is very important to determine which of the changes in the macular OCT are important for continuing treatment.

The different layers of the retina can be seen in the OCT, even though the images are not exactly anatomically equivalent. Figure 1 shows the different layers in a normal OCT [7] as well as the two different vascular plexuses that originate in the central artery [60].

The macular edema presents an increase in fluid accumulation in the central retinal layers, both intracellular and extracellular. The presence of an intracellular edema is difficult to observe in DME patients, and only in an edema that is secondary to acute central retinal artery occlusion can we see an increase in the width of ganglion cells and bipolar cell layers (Figure 3).

Depending on the location of extracellular fluid in the macular area, we can classify the macular edema. The standard clinical classification by Otani et al. [61] defines DME as follows:

1. Nontractional DME
   Spongiform-like
   Cystoid edema
   Serous retinal detachment
2. Tractional DME
   Tractional DME
   Epiretinal membrane DME

**Figure 4:**
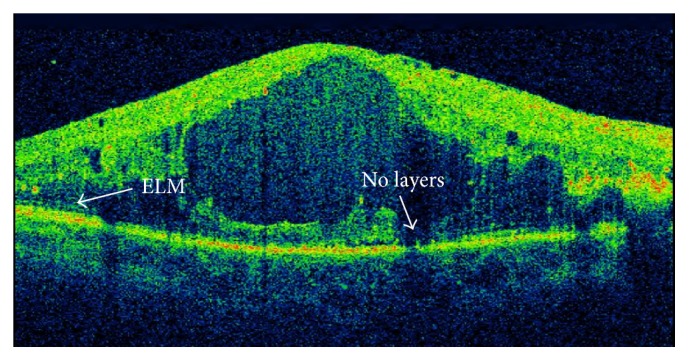
Absence of ellipsoid (ellipsoid zone) layer and external limiting membrane (ELM).

Although the retinal thickness is visible and, after treatment, this width improves and visual acuity increases, there is no direct relationship between retinal thickness and visual acuity [62]. In patients with DME, there is an increase in central retinal thickness, secondary to extracellular fluid, and a rupture in several retinal layers occurs, the most important of which being the ellipsoid layer and the ELM layer (Figure 4).

Visual function, determined by visual acuity and multifocal electroretinogram response, seems more related to the presence or absence of the different retinal layers. In particular, the presence or absence at the beginning of treatment of the ellipsoid layer and the ELM layer is highly related to final visual function (Figure 2), a finding which has been reported by authors of previous study [63]. 

**Figure 5:**
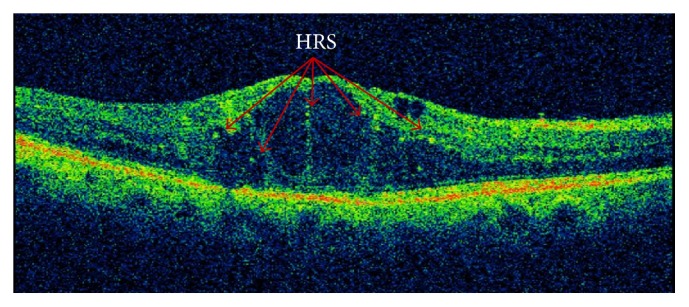
Cystoid diabetic macular edema with hyperreflective retinal spots.

*Can We Determine Inflammatory Changes Using the OCT?* A possible relationship between hyperreflective retinal spots (HRS) and inflammation in DME has recently been published [64]. Hyperreflective retinal spots (Figure 5) have been reported to predispose different lesions. Coscas et al. [65, 66] were the first to describe HRS as hyperreflective dots or foci, located in all retinal layers but with a predominant location in the outer retinal layers around the intraretinal cystoid spaces, and they suggested that this might be secondary to the activation of microglial cells [66]. Other possible explanations include that HRS are lipoprotein extravasations prior to hard exudates [67] or a degeneration of the photoreceptors or macrophages which engulf them [68]. An interesting study by Framme et al. [69] described the disappearance of HRS after anti-VEGF treatment in patients with DME and hypothesised that HRS represent a clinical marker of an inflammatory response, a finding supported by Coscas' observation that HRS represent clusters of microglia. Recently, Vujosevic et al. [70] in a case-control series in 20 eyes of 20 diabetic patients demonstrated that the decrease in HRS correlates with functional parameters, specifically retinal sensitivity.

Lastly, Vujosevic and Midena [71] demonstrated that HRS increase in patients with diabetes if they have DR. In the early stages, HRS are located in the same inner layers as the microglia. Later, the HRS are displaced to the outer layers, as happens with microglia when DR includes the outer retinal layers [72, 73]. An important finding of the study is that after anti-VEGF treatment, the baseline number of outer retinal HRS was linked to final visual acuity as well as ellipsoid zone disruption length and external limiting membrane disruption length [74]. In view of these similar results, more studies on the relationship between HRS, microglia, and DME are needed.

Another important image available from OCT is the presence of serious retinal detachment. This form of DME implies a detachment of the central foveal zone and is related to cystoid DME, which appears in 15% of cases of DME. The relationship with visual function is currently being debated, as some authors have not found a relationship with visual acuity [75]; others, however, have reported serious retinal detachment and large outer nuclear layer cysts as the two morphological changes that have the greatest negative impact on retinal function [76]. Retinal detachment can appear in the eyes as increased vascular permeability in the macula, which may be an important finding in terms of the possible existence of a diffused macular lesion. However, its presence is inversely related to the presence of ischemia, and patients with serious macular detachment do not present macular ischemia [77]. Curiously, serious macular detachment responds to intravitreal triamcinolone acetonide in direct proportion to the height of the serious macular detachment [78]—so, could this be a sign of inflammation? We can suppose that its appearance might be produced by a rupture in the external BRB due to a failure in the RPE pump function.

We can say, then, that the vasogenic or inflammatory etiology of DME is very difficult to distinguish from OCT. If we bear in mind that vasogenic changes appear at the beginning of the disease, then the spongiform-like macular edema might be due to a vasogenic cause and, in more advanced forms such as cystic or serious retinal detachment, inflammatory factors would be more likely. We do not know at the moment what is the pathophysiology of DME based on OCT.

In the future, the diagnosis of vasogenic or inflammatory etiology in DME will become increasingly important if we are to individualize treatments with either anti-VEGF or anti-inflammatory drugs or a combination of the two.

### 8.2. Fluorescein Angiography

Fluorescein angiography (FA) was first reported by Novonty and Alvis in 1961 [79], based on an injection of sodium fluorescein in aqueous solution into the vein. With a retinography, we can stimulate fluorescein with a source of white light passing through a filter that emits light at 480 nanometres, which is the wave that excites the molecules in the fluorescein. Then, we can use a barrier filter at 530 nanometres to visualise the fluorescence of the vascular bed. An important characteristic of fluorescein is that it binds with albumin, and the complex fluorescein-albumin cannot cross the blood-retinal barrier except in cases where the barrier has been ruptured, after which fluorescence in the retina is visible outside the vessels.

**Figure 6:**
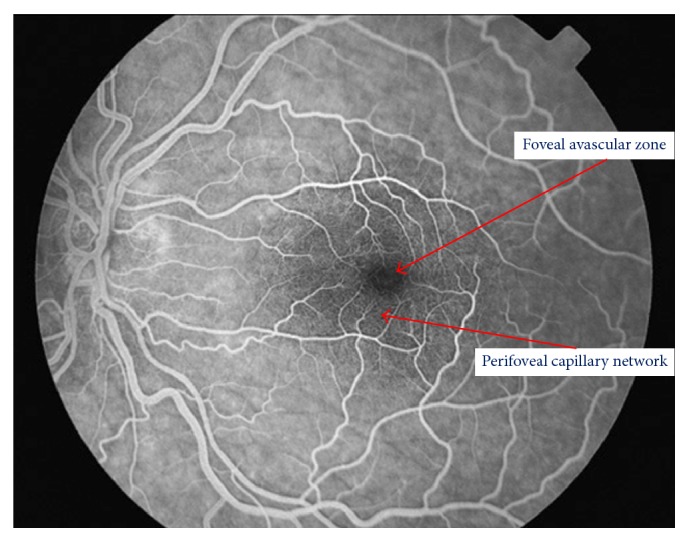
Normal fluorescein angiography showing the avascular foveal area and the perifoveal capillary network.

**Figure 7:**
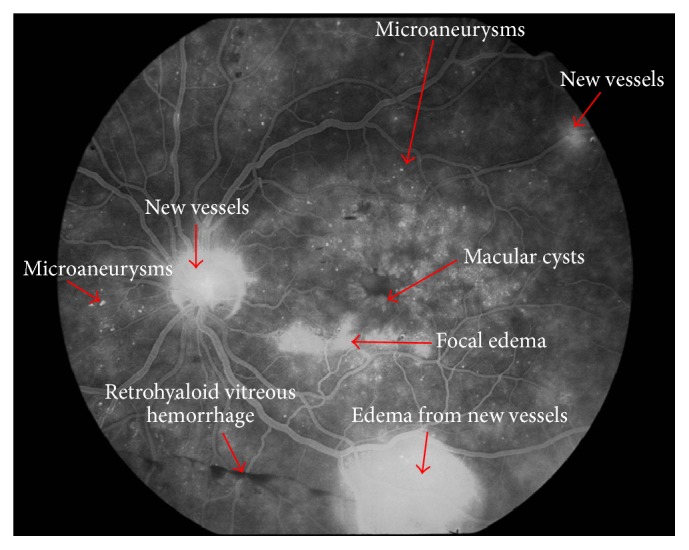
Fluorescein angiography of a patient with proliferative diabetic retinopathy. New vessels are visible in the optic disc and temporal (superior and inferior) arcades, and macular cysts and microaneurysms can be observed.

**Figure 8:**
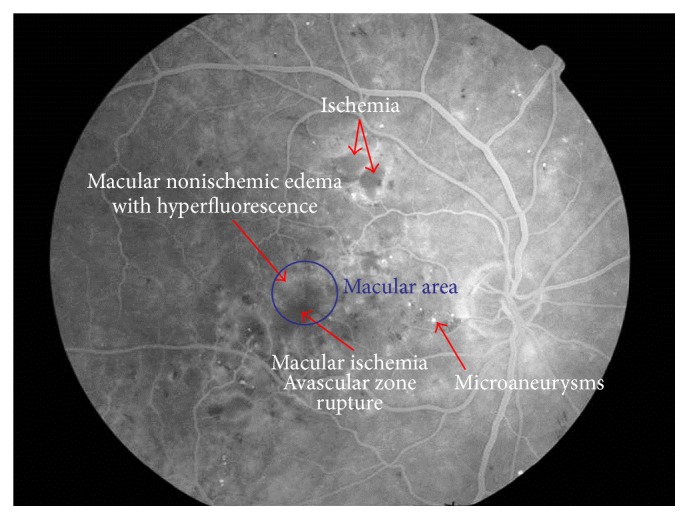
Fluorescein angiography of a patient with severe diabetic retinopathy and macular ischemia. We observe the rupture of avascular zone by ischemia and also hyperfluorescence area in macula at temporal side.

FA allows us to observe the retinal vasculature as well as how fluorescein crosses the arterial, capillary, and venous vascular phases (Figure 6). FA is an essential technique for determining a rupture in the inner-blood retinal barrier. In diabetes patients, we can see microaneurysms as dots of hyperfluorescence located at the capillary layer. The extravasation of fluorescein into the interstitial space and subsequent retinal edema helps us to diagnose DME. It also allows us to determine its origin in the vascular bed because of the incompetence of microaneurysms (focal macular edema) or as a diffuse extravasation in cases of diffuse macular edema (Figures 7 and 8). FA can also determine areas where there is no perfusion or retinal ischemia, which indicates a possible origin in cases of low visual acuity in patients with a normal macular area after treatment for DME. It is an important technique for determining the disruption of the internal blood-retinal barrier but does not allow us to determine the vasogenic or inflammatory etiology of DME [80, 81].

One problem with FA is that we have to inject fluorescein into the vein, which can have adverse effects, such as an allergic angioneurotic reaction in about 2% of cases and causing patient mortality (albeit extremely rare, occurring in only about 1 out of 220,000 angiographies).

A new technique has recently been added to the ophthalmic arsenal: OCT angiography (OCTA), which combines an image of the i-BRB vascular bed and an OCT of the macular area.

**Figure 9:**
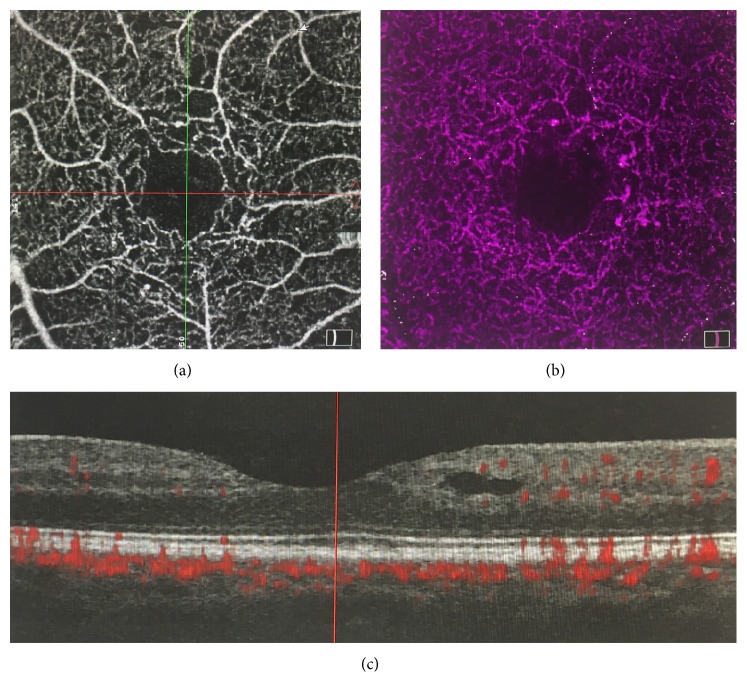
OCT angiography of a patient. (a) Superficial plexus, (b) inner plexus, and (c) optical coherence tomography.

OCTA gives us the opportunity to observe the vascular structure of the retinal vessels without an injection of fluorescein. OCTA can also determine the structure of the two vascular layers, or plexuses: the superficial or internal plexus layer, located between the optic nerve fiber layer and the ganglion cell layer; and the external or deep plexus, located in the inner plexiform layer (Figure 9).

OCTA allows us to observe macular vascularisation with special attention to the foveal avascular zone and the perivascular capillary bed. It is an important technique for determining the presence of ischemia in DR.

In patients with diabetes but without DR, it is important to observe an increase in the size of avascular foveal zone, which is a sign of the possible formation of microaneurysms: a stage which can be reversible if glycemic values are controlled. Also in the deep plexus, there are small areas of ischemia without DR, with a central enlargement of the avascular zone [82, 83].

In patients with developed DR, OCTA gives us an image of nonperfused areas, an increase in the anastomosis between the two plexuses, and larger capillary loops in the inner plexus [84, 85]. Initial neovascularisation is imaged as the presence of irregular thickened vessels that emerge from the surface of the retina or the optic disk.

One limitation of OCTA is that it is not a true fluorescein angiography because its hyperreflectivity is not equal to the hyperfluorescence of FA. In addition, OCTA images are limited to the macular area, so we cannot look at the vascular bed outside of the vascular temporal arcades. Finally, errors in the technique, such as using an 8 × 8 field, gives poor resolution, and surface artefacts or a mirroring effect in RPE can distort the images.

In the future, OCTA will help us to determine what happens in DR and allow us to treat more incipient lesions, but the technique needs more study and further development.

We can conclude that no examination technique helps us in the diagnosis of an inflammatory origin of DME; despite some orientation that can be extracted from the images, the presence of hyperreflective dots in OCT can be a sign of inflammatory changes and increased areas of hyperperfusion around the macula in FA lead us to think a vascular origin is present. Moreover, FA helps us to determine the hypoperfused areas by occlusions of the retinal vessels that can permit laser treatment of ischemic areas (if they are away from the macular area); if they are present in the macular area, we can determine a possible negative prognosis for visual recovery.

## 9. Conclusions

Diabetic macular edema (DME) is the leading cause of blindness in diabetic patients. Diagnosis is easy by exploring the retina under biomicroscopy and confirming it by OCT. Due to the efficacy of current treatments, it is essential to determine which etiology of macular edema is predominant.

Vasogenic changes secondary to hyperglycemia induce a rupture in the blood-retinal barrier (BRB), which begins the cascade of macular edema formation. However, the activation of a low-grade inflammation simultaneous to vasogenic changes will induce serious retinal damage and macular changes will become chronic.

Currently, DME is resistant to treatment in 30% of cases. The current anti-VEGF and steroid treatments are useful, but their use should be personalised. Prior knowledge of the predominant type of DME, vasogenic or inflammatory, is essential for determining the more effective type of drug.

## 10. Method of Literature Research

A PubMed and Web of Science search was conducted for the terms diabetic retinopathy, diabetic macular edema, oxidative stress, inflammation, blood-retina barrier, and optical coherence tomography in any publications between January 2000 and January 2015. A review of the abstracts identified relevant articles, which were later confirmed. The citations from these articles were also used to identify articles not found with the above search terms. Any non-English references were excluded.

## Figures and Tables

**Table 1:** Blood-retina barrier components, types of union, and the molecules located in retinal endothelial junctions.

Type of union	Molecule/component/cell
Tight junctions	Claudins (numbers: 1, 2, 5 and 12) Endothelial cell-specific adhesion molecule F11 receptor (jam-1) Junctional adhesions molecules (numbers: 2 and 3) Occludin Zonula occludens (numbers: 1 and 2)

Adherents junctions	*β*-Caterin VE-Cadherin N-Cadherin

Basal membrane	Basal membrane

Cells	Astrocytes Endothelial cells Microglia Müller cells Pericytes

## List of Abbreviations

AGEs: Advanced glycation end-products
BDNF: Brain-derived neurotrophic factor
CAV-1: Caveolin-1
CCL2: C-C motif ligand 2 (anteriorly named as monocyte chemoattractant protein-1 or MCP-1)
CRA: Central retina artery
DCCT: Diabetes Control and Complications Trial
DM: Diabetes mellitus
DR: Diabetic retinopathy
DRCR-net: Diabetic Retinopathy Clinical Research Network study
e-BRB: External-blood retina barrier
ELM: External limiting membrane layer
FasL: Fas-ligand
GCL: Ganglion cell layer
HIF-1*α*: Hypoxia-induced factor 1 *α*
i-BRB: Inner-blood retina barrier
ICAM-1: Inflammatory intercellular adhesion molecule-1
IL-6: Interleukin 6
INL: Inner nuclear layer
IOP: Intraocular pressure
IPL: Inner plexiform layer
IS/OS: Inner segment/outer segment layer, currently named ellipsoid zone
IVTA: Intravitreal doses of triamcinolone acetonide
MCP-1/CCL2: Monocytes quimio-attract 1, currently named as C-C motif ligand 2 (CCL2)
NFL: Nerve fiber layer
OCT: Optical coherence tomography
ONL: Outer nuclear layer
OPL: Outer plexiform layer
PDGF: Factor derived from platelets
PEDF: Pigmented epithelial derived factor
PKC: Protein kinase C
PRN: Pro re nata
RAGE: Receptors of advanced glycation end-products
RIDE/RISE: A study of ranibizumab injection in subjects with clinically significant macular edema (me) with centre involvement secondary to diabetes mellitus
RESOLVE: Safety and efficacy of ranibizumab in diabetic macular edema. A 12-month randomized, controlled, double-masked, multicentre phase II study.
RESTORE: A study of ranibizumab monotherapy or combined with laser monotherapy for diabetic macular edema
RN: Neurosensory retina
RPE: Retina pigment epithelium
STDR: Sight-threatening diabetic retinopathy
UKPDS: United Kingdom Prospective Diabetic Study
VA: Visual acuity
VEGF: Vascular endothelial growth factor
VIVID/VISTA: Intravitreal aflibercept for diabetic macular edema.
